# Global Research Trends on Colorectal Cancer (2014-2023): A Scientometric and Visualized Study

**DOI:** 10.34172/aim.31944

**Published:** 2024-10-01

**Authors:** Iman Menbari Oskouie, Hediyeh Alemi, Naghmeh Khavandgar, Heydar Ali Mardani-Fard, Azadeh AleTaha, Amir-Hossein Mousavian, Ali Rahimi, Mohammad Abdollahi, Akbar Soltani, Amir Kasaeian, Majid Sorouri

**Affiliations:** ^1^Urology Research Center, Tehran University of Medical Sciences, Tehran, Iran; ^2^Hematology, Oncology and Stem Cell Transplantation Research Center, Research Institute for Oncology, Hematology and Cell Therapy, Shariati Hospital, Tehran University of Medical Sciences, Tehran, Iran; ^3^Digestive Diseases Research Center, Digestive Diseases Research Institute, Shariati Hospital, Tehran University of Medical Sciences, Tehran, Iran; ^4^Department of Mathematics, Yasouj University, Yasouj, Iran; ^5^Evidence Based Medicine Research Center, Endocrinology and Metabolism Clinical Science Institute, Tehran University of Medical Sciences, Tehran, Iran; ^6^Endocrinology and Metabolism Research Center, Endocrinology and Metabolism Clinical Science Institute, Tehran University of Medical Sciences, Tehran, Iran; ^7^International Agriculture University, Tashkent, Uzbekistan; ^8^Liver and Pancreaticobiliary Research Center, Digestive Diseases Research Institute, Shariati Hospital, Tehran University of Medical Sciences, Tehran, Iran; ^9^Digestive Oncology Research Center, Digestive Diseases Research Institute, Shariati Hospital, Tehran University of Medical Sciences, Tehran, Iran; ^10^Research Center for Chronic Inflammatory Diseases, Shariati Hospital, Tehran University of Medical Sciences, Tehran, Iran; ^11^Clinical Research Development Unit, Shariati Hospital, Tehran University of Medical Sciences, Tehran, Iran

**Keywords:** Bibliometric, Colorectal cancer, Scientometric

## Abstract

**Background:**

Colorectal cancer (CRC) ranks as the third most common cancer worldwide, significantly contributing to cancer-related deaths and increasingly affecting younger populations. Although its impact on patients’ quality of life is profound, scientometric studies on CRC remain underexplored. The objective of this study was to evaluate the scientific literature on CRC from 2014 to 2023, employing a range of scientometric and statistical approaches.

**Methods:**

This study obtained CRC-related publications from the Scopus database. The analyses of the collaboration and co-occurrence among countries/regions, institutions, journals, references, authors, and keywords were conducted utilizing VOSviewer, facilitating the identification of key research trends and emergent subjects.

**Results:**

A review of Scopus entries yielded 200,385 papers on CRC in the last decade, noting a yearly increase in publications from 2014 to 2023. China emerged as the most prolific contributor with 46,674 documents. A positive correlation was identified between a country’s CRC research output and gross domestic product (GDP; *r*=0.961, *P*<0.001). The journal "Cancers" led to 3006 articles, and H. Brenner stood out as the foremost author with 452 publications. However, the Ministry of Education of the People’s Republic of China led institutional contributions to 3094 papers.

**Conclusion:**

With a leading count of 46674 articles, China dominated CRC research, particularly highlighted by the Ministry of Education of the People’s Republic of China. The primarily obtained keywords were CRC, cancer, prognosis, rectal cancer, and colon cancer. Despite the presence of global collaborations, there is a pressing need for increased research funding and support in the CRC, especially within developing nations. This study is a navigational tool for medical professionals, researchers, and surgical assistants to grasp the international progress and directions in CRC research.

## Introduction

 Colorectal cancer (CRC) is thought to be among the most prevalent types of cancer globally, with one to two million diagnosed each year. As a result, CRC is the third most prevalent cancer, with only lung, liver, and stomach cancers having higher rates of death. CRC ranks fourth in terms of causing cancer-related fatalities, resulting in 700 000 fatalities annually.^1^ From 1990 to 2012, there was a yearly increase in the occurrence of CRC, with Western countries accounting for 55% of the cases.

 Similar to other cancers, specific gene mutations are believed to contribute to the development of CRC. These mutations can occur in oncogenes, tumor suppressor genes, and genes involved in DNA repair mechanisms. Mutations are classified into three groups based on their origin (sporadic, inherited, and familial groups). Nevertheless, around 70% of CRC instances progress through a defined series of genetic mutations, resulting in the development of adenomas and, ultimately, carcinoma. Inherited forms of cancer contribute to just 5% of all CRC cases.^2^

 Age is a significant risk factor for CRC. The incidence of CRC before the age of 50 is rare, excluding inherited cases. However, the chance of developing CRC rises significantly after this age.^3^ Additionally, there are other permanent risk factors, such as a prior history of CRC or inflammatory bowel disease. Individuals with ulcerative colitis face a 3.7% increased risk of CRC, whereas those with Crohn’s disease have a 2.5% higher risk.^4,5^ Moderate physical activities have been shown to improve metabolic rates, gut motility, and long-term metabolic efficiency while reducing blood pressure.^6^ Smoking and alcohol consumption are also considered risk factors.^7^

 Currently, the primary treatment for CRC involves a comprehensive approach that considers both tumor-specific (e.g., metastasis, tumor growth rate, and biochemical markers) and patient-specific (e.g., health conditions and prognosis) factors.

 Scientometric and bibliometric analyses employ statistical tools to examine scientific publications. The realm of medical research has experienced a rise in scientometric studies owing to an increase in publication volume.^8-10^ Despite being a leading cause of mortality and its rising occurrence among the younger population, adversely impacting patient life quality, CRC has not been extensively explored through scientometric analysis. Accordingly, this study seeks to evaluate scientific articles on CRC from 2014 to 2023, using a combination of scientometric and statistical techniques.

## Materials and Methods

 The bibliographic information concerning CRC research was gathered from Scopus, a leading database for scholarly articles.^11^ The dataset compilation for this study was finalized on December 31, 2023. The collection process involved searching titles, abstracts, and keywords of publications from 2014 to 2023, based on predefined search criteria detailed in Supplementary file 1, resulting in a total of 200,365 records.

 The assembled reference database included comprehensive bibliographic data such as authors, publication titles, publication years, sources, volumes, issues, page numbers, affiliations, abstracts, keywords (both author-defined and indexed), references, document types, and more, which were subsequently processed through scientometric analysis.

 The statistical description and visualization of the data were performed using Microsoft Office Excel 2019 (Microsoft, Redmond, WA, USA). For more in-depth scientometric insights into the progression within these fields of research, the study engaged VOSviewer (version 1.6.16), a software known for its ability to visualize academic and scientific networks.^12,13^ This includes the representation of intricate relationships among authors, journals, collaborating nations, and citation patterns. VOSviewer also incorporates text mining to extract significant noun phrases from titles and abstracts, facilitating the creation of networks, clusters, and heatmaps to showcase research trends and connections.^12^

## Results

###  Overview of the Included Publications

**Figure 1:**
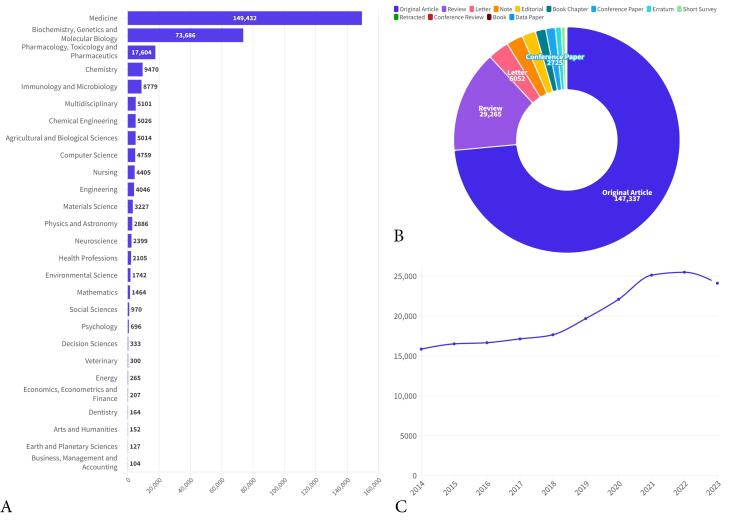
Number of Colorectal Cancer Publications From 2014 to 2023 in Different Subject Areas (1A), Article Types (1B), and Each Year (1C)

 The literature review in the Scopus database yielded 200,385 publications in the title-abstract-keyword fields related to CRC published between 2014 and 2023. The most popular subject areas of these publications were medicine (n = 149 432, 74.6%), biochemistry (n = 73 686, 36.8%), and pharmacology (n = 17 604, 8.8%), the details of which are shown in Figure 1A. The most frequent article types were original articles (n = 147 337, 73.5%), review articles (n = 29 265, 14.6%), and letters (n = 6052, 3.0%). The remaining publications were the other types of publications (note, editorial, book chapter, conference paper, erratum, short survey, retracted articles, conference review, book, and data paper), the data of which are provided in Figure 1B. Figure 1C likely depicts the distribution of published articles across different years. This visualization provides valuable insights into the trends and volume of research publications over time in the relevant field. In 2023, 24,118 articles were published, followed by 25 492 in 2022 and 25,131 in 2021.

###  Active Languages 

 The top 10 most frequent languages on CRC between 2014 and 2023 were English (n = 188 290, 94.0%), Chinese (n = 5063, 2.5%), Japanese (n = 2168, 1.15%), German (n = 1570, 0.78%), Spanish (n = 1153, 0.58%), French (n = 1043, 0.52%), Russian (n = 103, 0.51%), Czech (n = 265, 0.13%), Portuguese (n = 202, 0.10%), and Persian (n = 137, 0.07%).

###  Active Countries

**Figure 2:**
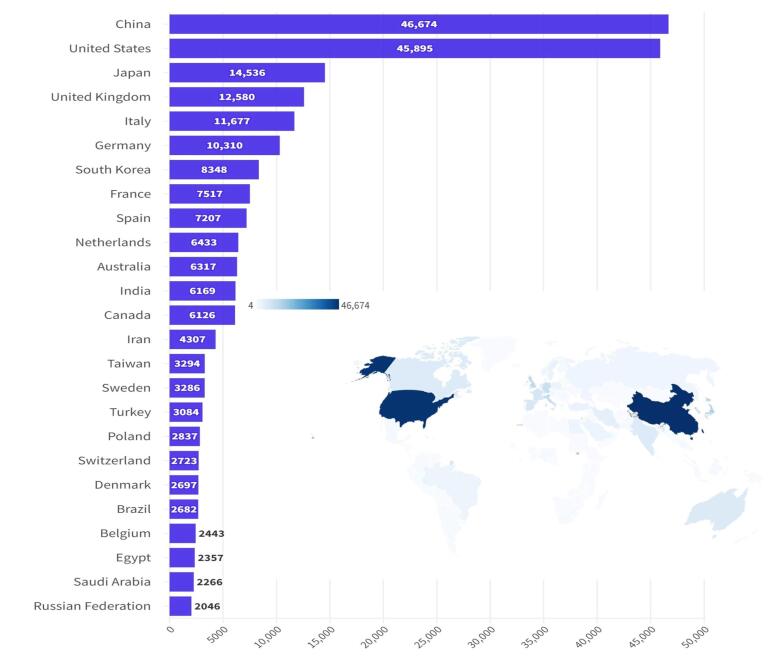
Number of Articles on Colorectal Cancer Produced by Different Countries From 2014 to 2023

Figure 2 illustrates the contribution of various countries to research on CRC from 2014 to 2023. These data can offer insights into the global landscape of CRC research and highlight the countries that are actively involved in studying this disease. The countries that ranked in the top 10 based on the highest number of published articles were China (n = 46 999), the USA (n = 46 027), Japan (n = 14 558), the UK (n = 12 596), Italy (n = 11 721), Germany (n = 10 334), South Korea (8380), France (n = 7540), Spain (n = 7228), and the Netherlands (n = 6451). Related data are presented in Table 1.

**Table 1:** Top 10 Productive Countries/Regions Associated with Colorectal Cancer

**Rank**	**Country**	**Documents**	**Percentage**	**TC**	**AAC**	**H-index**
1	China	46 999	23.46%	340 384	7.24	247
2	United States	46 027	22.97%	881 010	19.14	406
3	Japan	14 558	7.26%	193 826	13.31	171
4	United Kingdom	12 596	6.29%	348 539	27.67	253
5	Italy	11 721	5.85%	262 165	22.38	214
6	Germany	10 334	5.16%	251 507	24.34	209
7	South Korea	8380	4.18%	146 050	17.43	131
8	France	7540	3.76%	410 331	54.42	212
9	Spain	7228	3.61%	203 893	28.21	190
10	Netherlands	6451	3.22%	236 430	36.65	199

*Note*. TC: Total citations; AAC: Average article citations.

**Figure 3:**
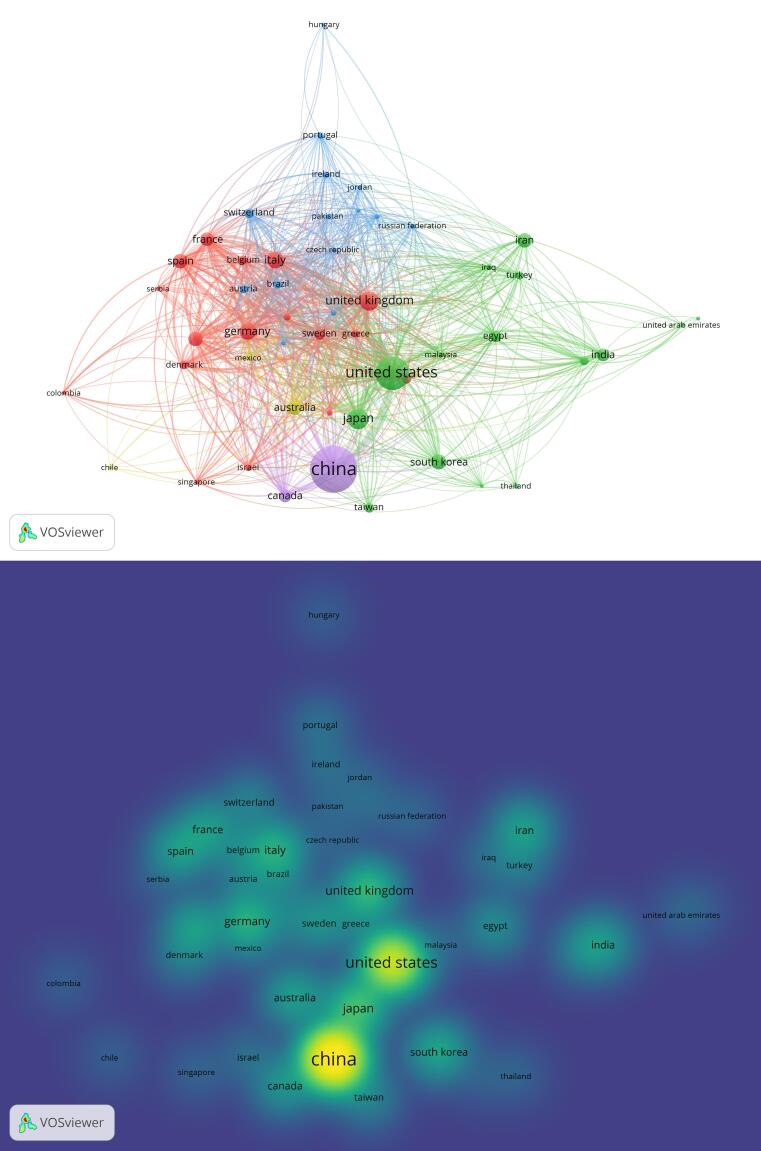
Analysis of Countries: Country Co-authorship Network Visualization Map (3A) and Country Co-authorship Density Visualization Map (3B)

 From the 160 countries that published studies on CRC, 135 met the criteria for inclusion in the clustering analysis (Figure 3A) by producing a minimum of ten articles and engaging in international author collaborations. The clustering analysis classified the countries into five distinct clusters denoted by colors (Cluster 1: red, Cluster 2: green, Cluster 3: blue, Cluster 4: yellow, and Cluster 5: purple). Moreover, the total link strength scores, reflecting the level of collaboration between countries, were computed. Figure 3C displays the international collaboration density map based on these scores.

###  Correlation Analysis 

 A strong and statistically significant positive correlation was observed between the number of articles published by countries on the CRC and their gross domestic product (GDP; r = 0.961, *P* < 0.001). Conversely, there was no notable correlation found between the number of articles produced by countries on CRC and their human development index or GDP per capita (*P* = 0.867 and *P* = 0.716, respectively).

###  Active Authors

**Figure 4:**
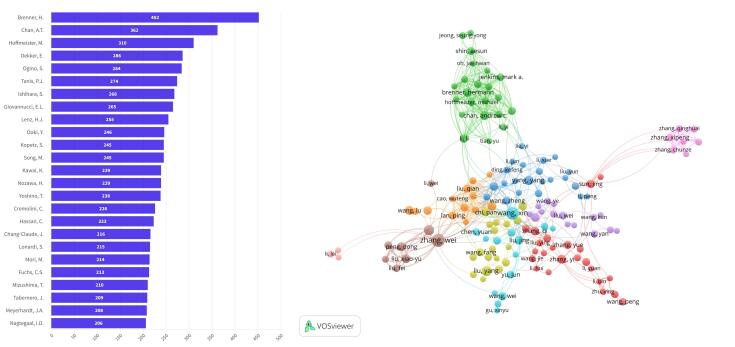
Analysis of Authors: Number of Articles on CRC Produced by the 25 Most Active Authors of Colorectal Cancer(4A) and Author Co-authorship Network Visualization Map (4B)

 The top 10 most active authors on CRC between 2014 and 2023 were Brenner (n = 448), Chan (n = 364), Hoffmeister (n = 309), Dekker (n = 289), Ogino (n = 284), Tanis (n = 274), Ishihara (n = 269), Giovannucci (n = 267), Lenz (n = 255), and Doki (n = 251) (Figure 4A and Table 2).

**Table 2:** The 10 Most Productive Authors with the Highest Number of Documents

**Rank**	**Author**	**Country**	**Documents**	**TC**	**AAC**	**H-index**
1	Brenner, H.	Germany	448	30 973	69.14	64
2	Chan, A. T.	China	364	14 340	39.39	61
3	Hoffmeister, M.	Germany	309	9948	32.19	52
4	Dekker, E.	Netherlands	289	9932	34.36	46
5	Ogino, S.	United States	284	15 934	56.1	66
6	Tanis, P. J.	Netherlands	274	9561	34.89	46
7	Ishihara, S.	Japan	269	4953	18.41	27
8	Giovannucci, E. L.	United States	267	13 443	50.35	59
9	Lenz, H. J.	United States	255	15 041	58.98	49
10	Doki, Y.	Japan	251	5329	21.23	36

*Note*. TC: Total citations; AAC: Average article citations.

 The clustering analysis included 160 authors who had published a minimum of 110 articles on CRC and had engaged in international collaboration among their co-authors (Figure 4B). This analysis led to the identification of ten distinct clusters associated with international collaboration.

###  Active Institutions

**Figure 5:**
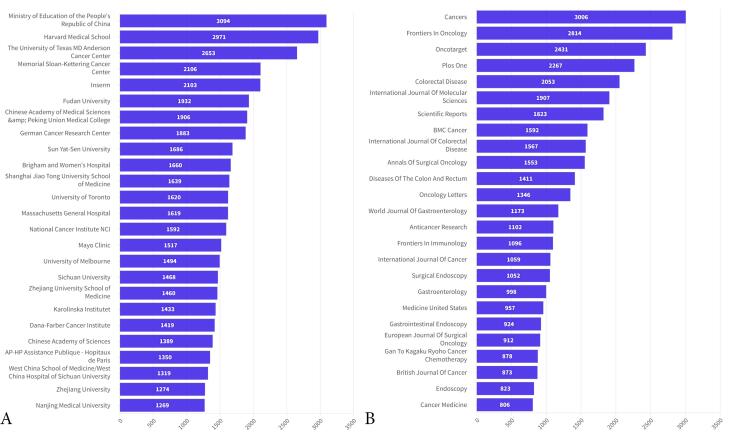
Number of Articles on Colorectal Cancer Produced by the 25 Most Active Institutions (5A) and Journals (5B)

 The top 10 institutions that generated the most volume of articles on CRC between 2014 and 2023 were the Ministry of Education of the People’s Republic of China (n = 3094), Harvard Medical School (n = 2971), the University of Texas MD Anderson Cancer Center (n = 2653), Memorial Sloan-Kettering Cancer Center (n = 2106), and Inserm (n = 2103). The remaining institutions were Fudan University (n = 1932), Chinese Academy of Medical Sciences and Peking Union Medical College (n = 1906), German Cancer Research Center (n = 1,883), Sun Yat-Sen University (n = 1686), and Brigham and Women’s Hospital (n = 1660) (Figure 5A).

###  Active Journals

 The top 116 journals published 300 or more articles on CRC between 2014 and 2023. The top 10 journals that printed the most significant amount of articles included Cancers (n = 3,006), Frontiers in Oncology (n = 2,814), Oncotarget (n = 2431), PloS One (n = 2267), colorectal disease (n = 2053), and International Journal of Molecular Sciences (n = 1907). The remaining journals were Scientific Reports (n = 1823), BMC Cancer (n = 1592), International Journal of Colorectal Disease (n = 1567), and Annals of Surgical Oncology (n = 1553) (Figure 5B).

###  Citation Analysis

 Out of the 200 385 articles on CRC, Table 3 lists the top 25 articles with the highest total citations. The average number of citations received per year by these articles is provided in the last column of Table 2.

**Table 3:** The Top 25 Most Cited Articles on Colorectal Cancer by Total Number of Citations

**No.**	**Article**	**Author**	**Journal**	**PY**	**TC**	**AC**
1	Global cancer statistics 2018: GLOBOCAN estimates of incidence and mortality worldwide for 36 cancers in 185 countries	Bray, F.	Cancer Journal for Clinicians	2018	58663	9778
2	Global cancer statistics 2020: GLOBOCAN estimates of incidence and mortality worldwide for 36 cancers in 185 countries	Sung, H.	Cancer Journal for Clinicians	2021	44900	14967
3	Global cancer statistics	Torre, L. A.	Cancer Journal for Clinicians	2015	24701	2745
4	Cancer incidence and mortality worldwide: Sources, methods, and major patterns in GLOBOCAN 2012	Ferlay, J.	International Journal of Cancer	2015	22580	2508
5	Cancer statistics, 2020	Siegel, R. L.	Cancer Journal for Clinicians	2020	14817	3704
6	Cancer statistics, 2017	Siegel, R. L.	Cancer Journal for Clinicians	2017	13615	1939
7	Cancer statistics, 2021	Siegel, R. L.	Cancer Journal for Clinicians	2021	13607	4535
8	Cancer statistics, 2018	Siegel, R. L.	Cancer Journal for Clinicians	2018	7133	1188
9	PD-1 blockade in tumors with mismatch-repair deficiency	Le, D. T.	New England Journal of Medicine	2015	6906	767
10	Global, regional, and national age-gender specific all-cause and cause-specific mortality for 240 causes of death, 1990-2013: A systematic analysis for the Global Burden of Disease Study 2013	Naghavi, M.	The Lancet	2015	5773	641
11	Global, regional, and national incidence, prevalence, and years lived with disability for 328 diseases and injuries for 195 countries, 1990-2016: A systematic analysis for the Global Burden of Disease Study 2016	Vos, T.	The Lancet	2017	5005	715
12	Projecting cancer incidence and deaths to 2030: The unexpected burden of thyroid, liver, and pancreas cancers in the United States	Rahib, L.	Cancer Research	2014	4844	485
13	Estimating the global cancer incidence and mortality in 2018: GLOBOCAN sources and methods	Ferlay, J.	International Journal of Cancer	2019	4843	969
14	Mismatch repair deficiency predicts response of solid tumors to PD-1 blockade	Le, D. T.	Science	2017	4443	634
15	Predictive correlates of response to the anti-PD-L1 antibody MPDL3280A in cancer patients	Herbst, R. S.	Nature	2014	3998	400
16	Cancer treatment and survivorship statistics, 2016	Miller, K. D.	Cancer Journal for Clinicians	2016	3922	490
17	UALCAN: A portal for facilitating tumor subgroup gene expression and survival analyses	Chandrashker, D. S.	Neoplasia	2017	3555	508
18	Global, regional, and national age-gender specific mortality for 264 causes of death, 1980-2016: A systematic analysis for the global burden of disease study 2016	Naghavi, M.	The Lancet	2017	3424	489
19	Detection of circulating tumor DNA in early- and late-stage human malignancies	Bettegowda, C.	Science Translational Medicine	2014	3330	333
20	MicroRNA therapeutics: Towards a new era for the management of cancer and other disease	Rupaimoole, R.	Nature Reviews Drug Discovery	2017	3316	474
21	Colorectal cancer statistics, 2017	Siegel, R. L.	Cancer Journal for Clinicians	2017	3247	464
22	Global patterns and trends in colorectal cancer incidence and mortality	Arnold, M.	Gut	2017	3129	447
23	The consensus molecular subtypes of colorectal cancer	Guinney, J.	Nature Medicine	2015	3098	344
24	Cancer treatment and survivorship statistics, 2019	Miller, K. D.	Cancer Journal for Clinicians	2019	3094	619
25	Colorectal cancer statistics, 2020	Siegel, R. L.	Cancer Journal for Clinicians	2020	3034	759

###  Trend Topics

 The most frequent words in CRC articles between 2014 and 2023 were human (n = 172 913), humans (n = 132 323), article (n = 115 629), CRC (n = 88 577), female (n = 85 599), and male (n = 84 347). Among these keywords, 160 were used in at least 6,600 different articles (Table 4). The most frequent author keywords were CRC (n = 6664), cancer (n = 1768), rectal cancer (n = 1456), colon cancer (n = 1352), prognosis (n = 1064), immunotherapy (n = 768), tumor microenvironment (n = 552), metastasis (n = 480), apoptosis (n = 472), chemotherapy (n = 440), and survival (n = 438).

**Table 4:** The 75 Most Frequently Used Keywords in Articles on Colorectal Cancer

**Keyword**	**Number of Uses**	**Keyword**	**Number of Uses**	**Keyword**	**Number of Uses**
Human	172 913	Review	26 265	Risk Factor	16 924
Humans	132 323	Retrospective Study	25 814	Neoplasm	16 619
Article	115 629	Protein Expression	24 298	Animal Experiment	16 578
Colorectal Cancer	88 577	Overall Survival	23 878	Signal Transduction	16 467
Female	85 599	Breast Cancer	23 268	Fluorouracil	16 380
Male	84,347	Cell Proliferation	22 825	Cancer Surgery	16 292
Controlled Study	68 187	Animals	22 060	Animal Model	15 729
Adult	65 389	Animal	21 376	Immunohistochemistry	15 596
Colorectal Neoplasms	57 712	Follow up	21 274	Cell Line, Tumor	15 458
Aged	56 366	Cancer Patient	21 268	Aged, 80, and Over	15 330
Pathology	52 796	Cancer Prognosis	21 143	Rectal Neoplasms	15 218
Middle Aged	50 706	Colonic Neoplasms	20 257	Tumor Cell Line	15 161
Priority Journal	49 255	Mouse	19 793	Lung Cancer	15 155
Major Clinical Study	48 798	Cancer Survival	19 682	Rectum Cancer	15 003
Colorectal Tumor	47 377	Prognosis	19 321	Metastasis	14 209
Genetics	37 721	Clinical Article	19 129	Carcinogenesis	13 806
Nonhuman	34 832	Neoplasms	18 737	Mortality	13 730
Metabolism	34 596	Retrospective Studies	18 447	Antineoplastic Activity	13 536
Unclassified Drug	34 074	Very Elderly	18 193	Prostate Cancer	13 480
Colon Cancer	32 107	Apoptosis	18 038	Tumor Marker	13 375
Human Cell	29 712	Cohort Analysis	18 026	Oxaliplatin	13 186
Antineoplastic Agent	28 851	Treatment Outcome	17 882	Upregulation	12 983
Human Tissue	28 809	Colonoscopy	17 294	Stomach Cancer	12 843
Cancer Staging	28 709	Colon Tumor	17 053	In Vitro Study	12 838
Procedures	27 579	Gene Expression	16 934	Cancer Chemotherapy	12 717

**Figure 6:**
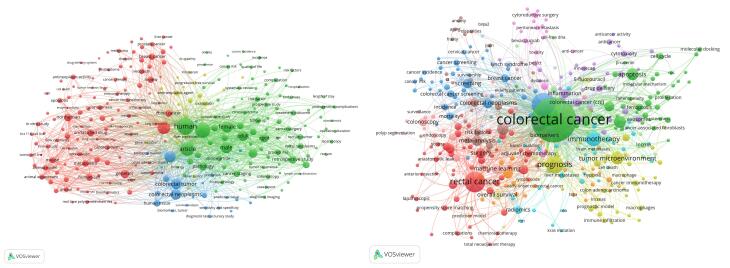
Network Visualization Map: Most Frequent Words (6A) and Most Frequent Author Keywords (6B)

Figure 6 shows the cluster network visualization map presenting the outcomes of the cluster analysis among these keywords.

## Discussion

 The review of article distribution related to CRC from 2014 to 2023 showed that, on average, 16 771 articles were published annually from 2014 to 2018 (range: 15 867 to 17 659). From 2019 to 2023, the yearly average of published articles rose to 23 324 (range: 19 689 to 25 492). In assessing the contributions by country, 16 of the 20 leading countries in CRC research were developed nations. Only four out of the top 20 countries (i.e., India, Iran, Taiwan, and Turkey) were classified as developing. It was apparent among the countries most active in CRC studies that there exists a significant association between a country’s research output and its GDP. This linkage implies that a country’s economic size and level of development predominantly determine its productivity in publishing CRC research.^14,15^

 The visualization of collaboration intensity through a density map, based on the total collaboration score among countries, highlighted the United States, England, Germany, Italy, France, the Netherlands, Spain, Australia, China, Sweden, Japan, and Canada as the countries engaging in the highest levels of partnership.

 Further examination of co-authorship networks for CRC research pointed out the crucial influence of geographic proximity on article production. The most collaborative clusters were identified as Canada and the United States; a group comprising China, Hong Kong, Macao, Singapore, and Taiwan; a European cluster with Austria, Belgium, France, Germany, Luxembourg, the Netherlands, and the United Kingdom; in addition, an Asian-Middle Eastern cluster included India, Indonesia, Iran, Iraq, Lebanon, Qatar, Saudi Arabia, South Korea, Thailand, and the United Arab Emirates.

 The leading journals for CRC research, ranked by the volume of articles published, included Cancers with 3006 articles, followed by Frontiers in Oncology (2814 articles), Oncotarget (2431), PLoS One (2267), Colorectal Disease (2053), International Journal of Molecular Sciences (1907), Scientific Reports (1823), and BMC Cancer (1592). The other journals were the International Journal of Colorectal Disease (1567) and the Annals of Surgical Oncology (1553). Researchers dedicated to CRC studies are advised to consider these journals for publishing their work.

 In terms of citations, the most cited CRC study was “Global cancer statistics 2018: GLOBOCAN estimates of incidence and mortality worldwide for 36 cancers in 185 countries” by Bray et al in the Cancer Journal for Clinicians,^16^ followed by “Global cancer statistics 2020: GLOBOCAN estimates of incidence and mortality worldwide for 36 cancers in 185 countries” by Sung et al, also in the same journal.^14^ The third most cited was “Global cancer statistics” by Torre et al, published in the Cancer Journal for Clinicians.^17^ It is recommended that those interested in CRC research consult these highly influential publications.

 This study is the latest bibliometric analysis on CRC, highlighting its novelty and significance. Previous notable works included those conducted by Wrafter et al, Darroudi et al, and Jin et al.^18-20^ Wrafter et al identified the top 100 most-cited CRC articles,^18^ and Darroudi et al and Jin et al performed bibliometric analyses on CRC treatment and the management of liver metastasis in CRC, respectively.^19,20^ Our literature review’s scope was bound by the choice of the Scopus database, which, while comprehensive, may omit some research found in the PubMed database, which lacks citation and co-citation analysis capabilities, and the Web of Science database, which focuses on higher-impact journals.^8-10^

## Conclusion

 A search within the Scopus database revealed a total of 200,385 documents focusing on “CRC” across title, abstract, and keyword fields from 2014 to 2023, with China leading in publication numbers (46 674 articles) and its Ministry of Education being the most prolific institution. The five most common keywords among these publications were ‘CRC’, ‘cancer’, ‘prognosis’, ‘rectal cancer’, and ‘colon cancer’. It is suggested that more research be performed to explore why CRC incidence rates are on the rise among young and middle-aged individuals. Despite the ongoing global collaborative efforts, there is a call for increased support and research into CRC, particularly in less developed nations. This paper aimed to inform clinicians, researchers, and surgical assistants about the international state of CRC research outcomes.

## Supplementary Files

Supplementary file 1. Searching Strategy.
