# Supplementary material for: Global Research Trends on Colorectal Cancer (2014-2023): A Scientometric and Visualized Study
Source: Arch Iran Med. 2024 Oct 1;27(10):563–72. doi: 10.34172/aim.31944 (PMC11532657; doi:10.34172/aim.31944)
Supplement: Supplementary file 1 — Searching Strategy. [file aim-27-563-s001.pdf]

Arch Iran Med. October 2024;27(10):563-S1

10.34172/aim.31944

Supplementary file 1. Searching Strategy

TITLE-ABS-KEY("Colorectal Neoplasm*" OR "Colorectal Cancer*" OR "Colorectal Tumor*" OR "Colorectal Carcinoma*" OR "Cancer Of The Colon" OR "Cancer Of Colon" OR "Proximal Colon Cancer*" OR "Anal Cancer*" OR "Anal neoplasm*" OR "Anus neoplasm*" OR "Cancer of the Anus" OR "Cancer of Anus" OR "Colon Cancer*" OR "Colorectal Cancer*" OR "Rectal Cancer*" OR "Rectal Tumor*" OR "Rectal Neoplasm*" OR "Rectal Neoplasia*" OR "Rectum cancer*" OR "Cancer of the Rectum" OR "Cancer of Rectum" OR "Anus Cancer*" OR "Carcinoma Of The Colon" OR "Tumor Of The Colon" OR "Anal Malignanc*" OR "Anal Carcinoma*" OR "Malignant Colonic Obstruction" OR "Neoplasm Of The Anus" OR "Colon cancer*" OR "Colon Adenocarcinoma" OR "Colorectal Cancer" OR "Colorectal Tumor*" OR "Colorectal Malignanc*" OR "Colorectal Neoplasm*" OR "Colorectal Neoplasia*" OR "Advanced Colorectal Neoplasia*" OR "Colorectal Neoplasia" OR "Transverse Colon Cancer*" OR "Transverse Colon Cancer*" OR "Transverse Colon Carcinoma*" OR "Transverse Colon Neoplasm*" OR "Descending Colon Cancer*" OR "Ascending Colon Cancer*" OR "Descending Colon Tumor*" OR "Ascending Colon Tumor*" OR "Left Sided Colonic Tumor*" OR "Ascending Colon Cancer*" OR "Sigmoid Colon Cancer*" OR "Sigmoid Colon Tumor*" OR "Adenocarcinoma of Sigmoid Colon" OR "Rectosigmoid Adenomas" OR "Cecum Cancer*" OR "Cancer of Cecum" OR "Cecal Tumor*" OR "Cecum Tumor*" OR "Cecal Malignanc*" OR "Carcinoma Of The Cecum" OR "Cecal Carcinoma*" OR "Cecal Neoplasm*" OR "Colon And Rectum Cancer*" OR "Rectum Cancer*" OR "Cancer Of The Colon And Rectum" OR "Rectal Tumor*" OR "Rectal Malignanc*" OR "Primary Rectal Malignanc*" OR "Rectal Malignant Tumor*" OR "Rectal Neoplasm*" OR "Rectal Neoplasia*" OR "Appendiceal Neoplasm*" OR "colonic neoplasm*" OR "colonic cancer*" OR "Sigmoid Neoplasm*" OR "Sigmoid Colon Neoplasm*" OR "Sigmoidal Cancer*" OR "Cancer of Sigmoid" OR "Cancer of the Sigmoid" OR "Rectal Tumor*" OR "Colorectal Neoplasms" OR "Colonic Neoplasms" OR "Sigmoid Neoplasms" OR "Colorectal Neoplasms, Hereditary Nonpolyposis" OR "Rectal Neoplasms" OR "Anus Neoplasms")
